# Usefulness of carotid duplex ultrasonography in predicting residual large-vessel occlusions after intravenous recombinant tissue plasminogen activator therapy in patients with acute ischemic stroke

**DOI:** 10.1007/s10396-022-01271-x

**Published:** 2022-12-04

**Authors:** Kei Kaburagi, Takahiro Shimizu, Yuta Hagiwara, Takayuki Fukano, Soichiro Shibata, Masashi Hoshino, Naoshi Sasaki, Hisanao Akiyama, Yasuhiro Hasegawa, Yoshihisa Yamano

**Affiliations:** 1 Department of Internal Medicine, Division of Neurology, St. Marianna University School of Medicine, 2-16-1 Sugao, Miyamae-Ku, Kawasaki, Kanagawa 216-8511 Japan; 2 Department of Neurology, Shin-Yurigaoka General Hospital, Kawasaki, Kanagawa Japan

**Keywords:** Carotid duplex ultrasonography, End-diastolic ratio, Large-vessel occlusions, Intravenous recombinant tissue plasminogen activator therapy

## Abstract

**Purpose:**

Endovascular therapy (EVT) preceded by intravenous thrombolysis with recombinant tissue plasminogen activator (iv-rtPA) has been established as a standard treatment in patients with stroke caused by large-vessel occlusion (LVO). Primary stroke centers without EVT competence need to identify patients with residual LVO after iv-rtPA therapy and transport them to an EVT-capable facility. Carotid ultrasonography (CUS) is easily applicable at bed side and useful for detecting extra- and intracranial LVO. This study aimed to determine whether CUS findings at admission are useful to predict patients with residual LVO after iv-rtPA.

**Methods:**

Patients scheduled to undergo iv-rtPA for acute cerebral infarction were registered. Before iv-rtPA, they underwent CUS, followed by CTA or MRA evaluation within 6 h after iv-rtPA. A model that can achieve 100% sensitivity for detecting residual LVO after iv-rtPA was studied.

**Results:**

This study included 68 of 116 patients treated with iv-rtPA during the study period. National Institutes of Health Stroke Scale (NIHSS) score (cutoff value = 10) on arrival, hyperdense MCA sign on non-contrast CT, end-diastolic (ED) ratio on CUS, and eye deviation were significantly different between patients with residual LVO after iv-rtPA and those without. If any of these clinical features are positive in the screening test, residual LVO could be predicted with 100% sensitivity, 50% specificity, 64% positive predictive value, and 100% negative predictive value.

**Conclusion:**

Prediction of residual LVO with 100% sensitivity may be feasible by adding CUS to NIHSS score > 10, the presence of eye deviation, and hyperdense MCA sign.

## Introduction

Endovascular therapy (EVT) preceded by intravenous recombinant tissue plasminogen activator (iv-rtPA) therapy has been proven to be effective in acute cerebral infarction [1–6]. In acute cerebral infarction, iv-rtPA is performed within 4.5 h from the onset of symptoms [1, 7], and EVT is performed within 6 h after symptom onset in patients with large-vessel occlusion (LVO) in the anterior circulation [8]. These treatments are expected to improve the outcome and functional prognosis. Of note, iv-rtPA should be started as early as possible because the therapeutic effect is highly time-dependent.

In patients with middle cerebral artery (MCA) occlusion, the recanalization rate after iv-rtPA was 51.7% at 6 h and 69.0% at 24 h [9]. Recanalization rates were lower in proximal occlusion. In patients with LVO in the anterior circulation, the recanalization rate after a median of 7 h was only 1/3 of that in patients treated with iv-rtPA alone [2]. Thus, the effects of iv-rtPA treatment alone for patients with LVO are limited. Randomized trials (MR CLEAN, ESCAPE, EXTEND-IA, SWIFT PRIME, and REVASCAT) comparing iv-rtPA therapy with EVT preceded by iv-rtPA therapy in patients with LVO demonstrated that adding EVT to iv-tPA therapy was effective [2–6]. In 2015, the American Heart Association/American Stroke Association (AHA/ASA) guidelines raised the evidence for EVT to class 1, where patients with internal carotid artery (ICA) and M1 occlusion are recommended to start EVT within 6 h [8].

Diagnosis of LVO (ICA or MCA M1 occlusion) is essential to determine the indication for EVT. Initial imaging of acute stroke should include non-contrast computed tomography (NCCT) or magnetic resonance imaging (MRI) to rule out intracranial hemorrhage, followed by CT angiography (CTA) or MR angiography (MRA) to evaluate extra- and/or intracranial LVO. Primary stroke centers (PSCs) have been reported to more likely provide iv-rtPA therapy and improve outcomes after acute cerebral infarction [10, 11]. However, PSCs without EVT competence are required to diagnose LVO at an early stage and transfer promptly to EVT-capable facilities (drip-and-ship strategy). The time to EVT after iv-rtPA is an important determinant of recanalization rate and functional outcome; the shorter the door-to-puncture time (D2P), the better the outcome of EVT. A target time frame such as D2P ≤ 120 min has been set, and the number of patients with favorable outcomes has increased [12]. Although NCCT has been widely used for iv-rtPA therapy, vascular imaging with CTA or MRA is needed to identify patients eligible for EVT. Such further vascular imaging may delay the transport to an EVT-capable facility.

Carotid ultrasonography (CUS) is a useful tool to detect LVO in the anterior circulation using the end-diastolic (ED) ratio of the common carotid end-diastolic flow velocity. The ED ratio is ≥ 1.4 in embolic or thrombotic occlusion of the ICA [13]. In cardiogenic cerebral embolism alone, the ED ratio is reportedly ≥ 4.0 in ICA occlusion and 1.3 ≤ ED ratio < 4.0 in MCA occlusion [13, 14]. Therefore, we hypothesized that if the ED ratio before iv-rtPA is related to the prediction of residual LVO (rLVO) after iv-rtPA, it can be applied to rapid ‘drip-and-ship’ transport after iv-rtPA. In this study, we aimed to verify whether neurologic findings, NCCT findings of the head, and CUS findings can predict rLVO after iv-rtPA in patients with acute cerebral infarction.

## Patients and methods

We performed a single-center retrospective study. Patients who were admitted to our hospital between November 1, 2010 and December 31, 2020, diagnosed with acute cerebral infarction based on the initial NCCT scan within 4.5 h of symptom onset, and subsequently underwent iv-rtPA were enrolled in this study. In particular, we included those who underwent CUS before iv-rtPA and in whom CTA or MRA was performed within 6 h after the end of iv-rtPA infusion. We also retrospectively examined each patient’s history of vascular risk (hypertension, diabetes mellitus, and dyslipidemia), lesions on MRI and CT scan, and presence or absence of intracranial major artery occlusion on MRA and CTA. We defined LVO as a proximal lesion corresponding to the ICA and the horizontal portion of the MCA (M1). MRI/MRA was conducted using a 1.5 T Visart/Excelart (Toshiba Medical Co., Ltd., Japan), and the intracranial large vessel was imaged and evaluated by Time of Flight. Two experienced stroke specialists who were blinded to the patient’s clinical information evaluated LVO lesions in all patients. CTA was performed using a Siemens 64-row (128 slices) multi-slice CT system with a bolus tracking technique.

We surveyed risk factors for atherosclerosis such as hypertension, diabetes mellitus, and dyslipidemia. Hypertension was defined as systolic blood pressure ≥ 140 mmHg or diastolic blood pressure ≥ 90 mmHg or taking antihypertensive drugs. Diabetes mellitus was defined as fasting blood glucose ≥ 126 mg/dL or casual blood glucose ≥ 200 mg/dL and HbA1c ≥ 6.5% during hospitalization (initial diagnosis), or undergoing oral hypoglycemic or insulin therapy. Lastly, dyslipidemia was defined as LDL cholesterol ≥ 140 mg/dL or triglycerides ≥ 150 mg/dL or HDL cholesterol ≤ 40 mg/dL or taking medication for dyslipidemia.

We measured the end-diastolic flow velocity of the right and left common carotid arteries using an Xalio (Toshiba Medical Co., Ltd., Japan) with a 7.5-MHz linear probe and calculated the end-diastolic flow velocity ratio. Patients were divided into three groups based on the end-diastolic flow velocity ratio according to previous reports: ≥ 4.0, ≥ 1.4 and < 4.0, and < 1.4 [13, 14]. All patients were measured at 1 to 2 cm from the carotid sinus on the trunk side. The sample volume was 2 of 3 of the vessel diameters and the Doppler angle of incidence was 60° [15]. The examiners were the initial care providers of each patient, all of whom were neurologists engaged in stroke care and experienced in performing CUS. The intra-observer coefficient of variance (CV) % was 3.00%, and inter-observer CV% was 4.81%.

Statistical data were analyzed using the statistical analysis software SPSS (ver. 26 for Windows; IBM SPSS Statistics, Chicago, USA). We used *t*-test or Mann–Whitney *U* test for between-group comparison of continuous variables and *χ*^2^ test for the prevalence of risk factors. A *P* value < 0.05 was considered significant. Moreover, factors that might predict rLVO after iv-rtPA were evaluated retrospectively from medical records. These factors included the National Institutes of Health Stroke Scale (NIHSS) score on arrival, history of vascular risk, presence or absence of eye deviation, aphasia (the evaluation items of the Emergent Large-vessel Occlusion [ELVO] screen [16]), CT findings, and CUS findings. We also calculated the sensitivity, specificity, positive predictive value (PPV), and negative predictive value (NPV) of a prediction method that aimed to extract as high as 100% of rLVO after iv-rtPA.

## Results

**Fig. 1:**
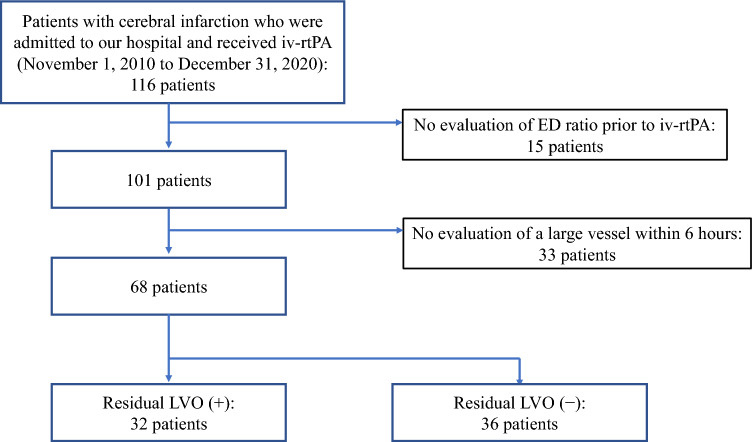
Trial flow diagram of this study. *iv-rtPA* intravenous recombinant tissue plasminogen activator, *ED* end-diastolic, *rLVO* residual large vessel occlusion

Out of 116 patients treated with iv-rtPA during the observation period, 48 were excluded because of no evaluation of the ED ratio before iv-rtPA (*n* = 15) and no evaluation of an intracranial large vessel within 6 h after iv-rtPA (*n* = 33). Ultimately, 68 patients (35 men; mean age, 73.4 years) were included in the analysis (Fig. 1). The time from iv-rtPA to vascular evaluation after intravenous infusion was 115.6 ± 110.8 min (7–360 min), and 32 patients had rLVO. Patients with rLVO after iv-rtPA had a significantly higher NIHSS score than those without (18.7 ± 6.8 *vs.* 10.5 ± 5.7, *P* < 0.001) (Table 1). In addition, the presence of a hyperdense MCA sign on NCCT at admission, abnormal ED ratio (≥ 1.4), and eye deviation were significantly different between the two patient groups (*P* = 0.047, 0.013, and 0.008, respectively). There was no significant association between rLVO and stroke subtypes.

**Table 1:** Patient characteristics

	Total number of eligible patients *N* = 68	rLVO ( −) *N* = 36	rLVO ( +) *N* = 32	*P* value
Age (years old) mean ± SD	73.4 ± 12.5	71.3 ± 14.5	75.8 ± 9.4	0.406
Male, *n* (%)	35 (51.5)	17 (47.2)	18 (56.3)	0.457
Hypertension, *n* (%)	37 (57.4)	17 (55.6)	19 (59.4)	0.751
Diabetes, *n* (%)	13 (19.1)	9 (25.0)	4 (12.5)	0.191
Dyslipidemia, *n* (%)	30 (45.6)	16 (44.4)	15 (46.9)	0.841
Atrial fibrillation, *n* (%)	28 (41.2)	12 (33.3)	16 (50.0)	0.163
NIHSS score at admission	**14.3 ± 7.4**	**10.5 ± 5.7**	**18.7 ± 6.8**	** < 0.001**
Consciousness (JCS ≥ 3), *n* (%)	37 (54.4)	17 (47.2)	20 (62.5)	0.207
Eye deviation, *n* (%)	**25 (36.8)**	**8 (22.2)**	**17 (53.1)**	**0.008**
Stroke subtypes				0.107
LI	7 (10.3)	7 (19.4)	0	
ATBI	7 (10.3)	3 (8.3)	4 (12.5)	
CE	34 (50.0)	17 (47.2)	17 (53.1)	
Undetermined	14 (20.6)	7 (19.4)	7 (21.9)	
Other causes	6 (8.8)	2 (5.6)	4 (12.5)	
Aphasia, *n* (%)	34 (50.0)	18 (50.0)	16 (50.0)	1.000
Hyperdense MCA sign, *n* (%)	**16 (23.5)**	**5 (15.6)**	**11 (34.4)**	**0.047**
ED ratio before iv-rtPA				**0.013**
ED ratio < 1.4	**34 (50.0)**	**24 (66.7)**	**10 (31.3)**	
1.4 ≤ ED ratio < 4.0	**21 (30.9)**	**8 (22.2)**	**13 (40.6)**	
4.0 ≥ ED ratio	**13 (19.1)**	**4 (11.1)**	**9 (28.1)**	
D-dimer (µg/mL)	2.56 ± 3.83	1.89 ± 2.86	3.31 ± 4.63	0.189

Results with significant differences were in bold

*rLVO* residual large-vessel occlusion, *NIHSS* National Institutes Of Health Stroke Scale, *LI* lacunar infarction, *ATBI* atherothrombotic brain infarction, *CE* cardioembolism, *ED*: end-diastolic, *iv-rtPA* intravenous recombinant tissue plasminogen activator

**Fig. 2:**
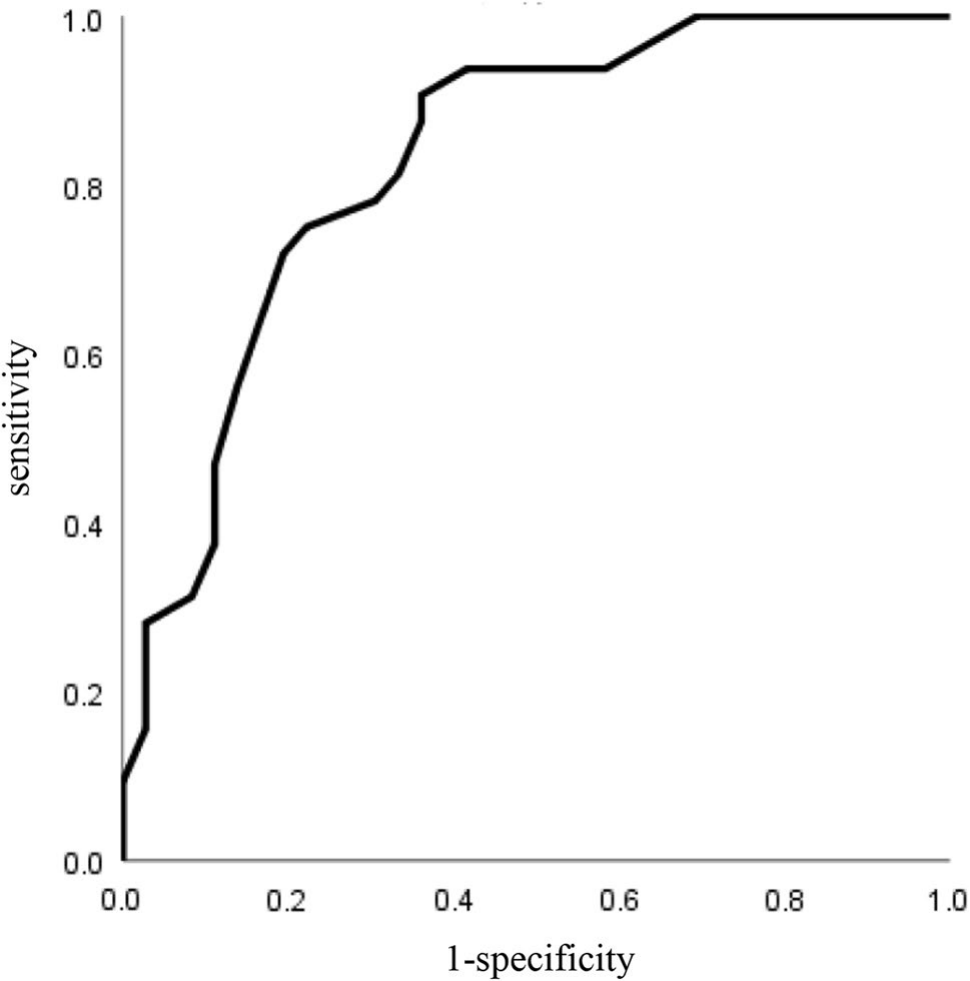
The receiver-operating characteristic curve of NIHSS scores predicting rLVO. *NIHSS* National Institutes of Health Stroke Scale, *rLVO* residual large-vessel occlusion

**Fig. 3:**
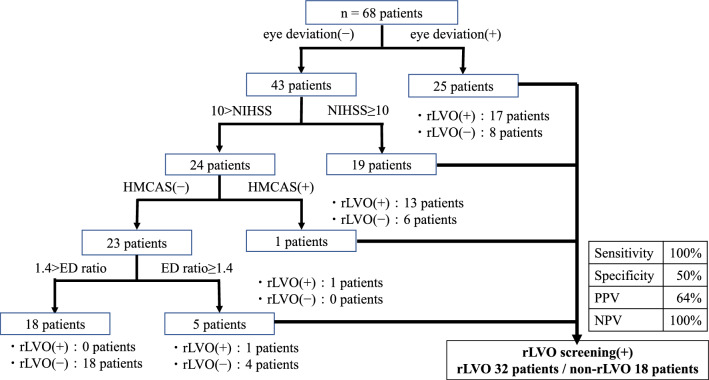
Algorithm for raising the predictive sensitivity. *rLVO* residual large-vessel occlusion, *NIHSS* National Institutes Of Health Stroke Scale, *ED* end-diastolic, *HMCAS* hyperdense middle cerebral artery sign, *PPV* positive predictive value, *NPV* negative predictive value

To determine the cutoff value of NIHSS score to predict rLVO, ROC curve analysis was constructed using NIHSS score, and the area under the curve (AUC) was 0.83. The optimal NIHSS score cutoff value obtained using the Youden index was 10 (Fig. 2, Table 2). According to these results, when a positive rLVO screening test is defined as NIHSS score ≥ 10, ED ratio on CUS ≥ 1.4, presence of hyperdense MCA sign, or presence of eye deviation, rLVO cases can be identified with 100% sensitivity, 50% specificity, 64% PPV, and 100% NPV (Fig. 3). When CUS findings were not used, one LVO case did not show any abnormal findings in terms of NIHSS score, hyperdense MCA sign, or eye deviation, and was thereby omitted from screening.

**Table 2:** Accuracy of NIHSS score for prediction of residual large-vessel occlusion

	NIHSS score
Cutoff point	≥ 10
Sensitivity	0.906
Specificity	0.639
AUC	0.830

*NIHSS* National Institutes Of Health Stroke Scale, *AUC* area under the curve

## Discussion

In this study, we aimed to clarify whether the CUS ED ratio evaluated before iv-rtPA can be used to predict rLVO after iv-rtPA using various factors. The present study showed that high NIHSS score on arrival, hyperdense MCA sign on CT scan, ED ratio ≥ 1.4, and eye deviation were associated with rLVO. Combining these findings, rLVO could be predicted with a sensitivity of 100% without a single missed case, at least in this cohort.

Eye deviation generally suggests the presence of cortical lesions and is known to be associated with LVO [17]. However, eye deviation is not observed exclusively in LVO as it can also occur with focal damage of the frontal eye field or paramedian pontine reticular formation (PPRF). In this study, 8 of 25 patients with eye deviation were negative for rLVO. Thus, the sensitivity and specificity of eye deviation alone were 53.1% and 77.8%, respectively, suggesting that eye deviation alone was not enough to predict LVO. Although symptoms of aphasia, suspected presence of cortical lesions, showed no significant difference in this study, this may be due to insufficient assessment using the aphasia items of NIHSS because of a slight disturbance in consciousness.

Patients with LVO have generally high NIHSS scores [18]. While the NIHSS is one of the best tools for predicting the presence of LVO in the emergency scene, it is not sufficiently accurate in patients with an NIHSS score of ≥ 10 (sensitivity, 73%; specificity, 74%) and in patients with an NIHSS score of ≥ 6 (sensitivity, 87%; specificity, 52%) [19]. It has also been reported that LVO is found in 18% of patients with NIHSS scores of 0–4 and 39% of patients with NIHSS scores of 5–8, and approximately half of patients with LVO have an NIHSS score of < 10. The sensitivity of NIHSS for diagnosing the presence of LVO has been reported to be not necessarily high [20]. In this study, 3 of 32 patients (9.4%) with rLVO after iv-rtPA had an NIHSS score of < 10. Furthermore, 13 of 42 patients with an NIHSS score of ≥ 10 were negative for rLVO (data not shown). Thus, the sensitivity and specificity of NIHSS alone to predict rLVO were 90.6% and 63.9%, respectively.

In this study, a hyperdense MCA sign [21], which is the high CT absorption value of thrombi in M1 vessels on non-contrast CT, was also shown to be associated with the presence of rLVO (Table 1). However, as many as 15.6% of patients without LVO had a hyperdense MCA sign in this study (data not shown), and the sensitivity and specificity of hyperdense MCA sign alone were 34.4% and 86.1%, respectively. The causes of false-positive cases are reported to include vessel wall calcifications, high hematocrit levels, and edematous changes in the brain around the MCA [22]. Although the sensitivity and specificity of a hyperdense MCA sign alone are not enough to predict rLVO, it may be reasonable to transfer patients with a hyperdense MCA sign to EVT-capable facilities, because it has been reported to be a factor of a poor outcome after intravenous thrombolytic therapy [23–25].

A high ED ratio (≥ 1.4) before iv-rtPA was also associated with the presence of rLVO (Table 1). However, 10 of 32 patients (31.3%) with rLVO after iv-rtPA had an ED ratio < 1.4, indicating that the presence of rLVO cannot be determined based on ED ratio alone. Furthermore, the ED ratio is known to be difficult to assess in patients with bilateral lesions, well-developed collateral circulation, and severe aortic regurgitation [14]. In this study, the sensitivity and specificity of ED ratio alone were 68.8% and 69.4%, respectively, neither of which is high, but there was one patient out of 32 (3.1%) in the study population for whom ED ratio was the only item to screen for rLVO.

Although each of the four factors alone was insufficient to predict rLVO, it was shown that all rLVO patients could be identified using the criterion of being positive for any of the four factors (Fig. 3), suggesting that a combination of four factors can increase the sensitivity and allow the transfer of patients without performing CTA or MRA. Importantly, there were four patients with LVO who underwent recanalization after iv-rtPA (assigned to the non-rLVO group in this study), and these patients could also be identified based on a combination of four factors (one patient with eye deviation and ED ratio ≥ 1.4, three patients with NIHSS score ≥ 10 and ED ratio ≥ 1.4). To improve the prognosis of ischemic stroke patients, it is necessary to detect all patients with possible rLVO and transfer them to a facility where EVT is possible in PSCs where EVT cannot be performed. Meanwhile, overestimation with low specificity may lead to the heavy workload of EVT-capable facilities. However, considering that the median time required to perform additional CTA or MRA was 65 min in our cohort (data not shown), and that the ideal time from presentation to EVT puncture is within 2 h [13], we believe that saving time should be prioritized over adding CTA or MRA to increase specificity. It will be important to find more factors to increase the specificity without wasting time. Furthermore, our data suggest the possibility that the use of prehospital CUS ED ratio evaluation by ambulance crews would be useful to increase the number of patients suspected of having LVO who can be directly transferred to EVT-capable facilities. This hypothesis should be tested in the future study.

This study had several limitations. First, the sample size was small because only iv-rtPA cases at a single institution were studied. Second, the vascular evaluation time after iv-rtPA varied from case to case (7–360 min after ivt-PA). This may have been due to the low priority of LVO evaluation during the period before evidence of EVT efficacy was demonstrated. Third, the definition of LVO was limited to ICA to M1 (exclusion of posterior circulation occlusion and occlusion beyond M2). Finally, the examiner of CUS varied from patient to patient. Recently, many studies have been conducted to investigate patients with LVO who are directly treated with EVT without iv-rtPA. However, medical services for stroke in underpopulated areas remain unestablished, and patients need to be transported to a nearby PSC or a similar primary care facility. Nevertheless, CUS can be easily evaluated even by non-stroke specialists. Hence, acquisition of examination techniques and addition of findings as indexes may help us perform the drip-and-ship approach appropriately.

## Conclusion

The addition of CUS to the initial evaluation of patients with acute cerebral infarction can increase the sensitivity of predicting rLVO after iv-rtPA. Prediction of residual LVO with 100% sensitivity may be feasible by adding CUS to NIHSS score > 10, presence of eye deviation, and hyperdense MCA sign.

## Data Availability

Raw data were generated at St. Marianna University School of Medicine. Derived data supporting the findings of this study are available from the corresponding author [K.K] on request.

## Abbreviations

AUC: Area under the curve
CT: Computed tomography
NCCT: Non-contrast computed tomography
CUS: Carotid ultrasonography
ED: End-diastolic
ICA: Internal carotid artery
LVO: Large-vessel occlusion
rLVO: Residual large-vessel occlusion
MCA: Middle cerebral artery
MRA: Magnetic resonance angiography
MRI: Magnetic resonance imaging
NIHSS: National Institutes of Health Stroke Scale
NPV: Negative predictive value
PPV: Positive predictive value
PSC: Primary stroke centers
