# Correction: Usefulness of carotid duplex ultrasonography in predicting residual large-vessel occlusions after intravenous recombinant tissue plasminogen activator therapy in patients with acute ischemic stroke

**DOI:** 10.1007/s10396-025-01549-w

**Published:** 2025-05-13

**Authors:** Kei Kaburagi, Takahiro Shimizu, Yuta Hagiwara, Takayuki Fukano, Soichiro Shibata, Masashi Hoshino, Naoshi Sasaki, Hisanao Akiyama, Yasuhiro Hasegawa, Yoshihisa Yamano

**Affiliations:** 1 Department of Internal Medicine, Division of Neurology, St. Marianna University School of Medicine, 2-16-1 Sugao, Miyamae-Ku, Kawasaki, Kanagawa 216-8511 Japan; 2 Department of Neurology, Shin-Yurigaoka General Hospital, Kawasaki, Kanagawa Japan

**Correction: Journal of Medical Ultrasonics 50:103–109 (2023)** 10.1007/s10396-022-01271-x

The article “Usefulness of carotid duplex ultrasonography in predicting residual large‑vessel occlusions after intravenous recombinant tissue plasminogen activator therapy in patients with acute ischemic stroke”, written by Kei Kaburagi, Takahiro Shimizu, Yuta Hagiwara, Takayuki Fukano, Soichiro Shibata, Masashi Hoshino, Naoshi Sasaki, Hisanao Akiyama, Yasuhiro Hasegawa and Yoshihisa Yamano, was originally published under exclusive license to The Author(s), under exclusive licence to The Japan Society of Ultrasonics in Medicine. As a result of the subsequent decision to publish the article under the open access model, the article’s copyright notice was changed on 4 April 2025 to © The Author(s) 2022 and the article is now distributed under a Creative Commons Attribution 4.0 International License, which permits use, sharing, adaptation, distribution and reproduction in any medium or format, as long as you give appropriate credit to the original author(s) and the source, provide a link to the Creative Commons licence, and indicate if changes were made. The images or other third party material in this article are included in the article’s Creative Commons licence, unless indicated otherwise in a credit line to the material. If material is not included in the article’s Creative Commons licence and your intended use is not permitted by statutory regulation or exceeds the permitted use, you will need to obtain permission directly from the copyright holder. To view a copy of this licence, visit http://creativecommons.org/licenses/by/4.0.

The original article has been corrected.

